# The Applications of Single-Cell RNA Sequencing in Atherosclerotic Disease

**DOI:** 10.3389/fcvm.2022.826103

**Published:** 2022-02-08

**Authors:** Lotte Slenders, Daniëlle E. Tessels, Sander W. van der Laan, Gerard Pasterkamp, Michal Mokry

**Affiliations:** ^1^Central Diagnostics Laboratory, University Medical Center Utrecht, University Utrecht, Utrecht, Netherlands; ^2^Laboratory of Experimental Cardiology, Department of Cardiology, University Medical Center Utrecht, University Utrecht, Utrecht, Netherlands

**Keywords:** scRNA-sequencing, transcriptomics, -omics, single-cell, atherosclerosis

## Abstract

Atherosclerosis still is the primary cause of death worldwide. Our characterization of the atherosclerotic lesion is mainly rooted in definitions based on pathological descriptions. We often speak in absolutes regarding plaque phenotypes: vulnerable vs. stable plaques or plaque rupture vs. plaque erosion. By focusing on these concepts, we may have oversimplified the atherosclerotic disease and its mechanisms. The widely used definitions of pathology-based plaque phenotypes can be fine-tuned with observations made with various -omics techniques. Recent advancements in single-cell transcriptomics provide the opportunity to characterize the cellular composition of the atherosclerotic plaque. This additional layer of information facilitates the in-depth characterization of the atherosclerotic plaque. In this review, we discuss the impact that single-cell transcriptomics may exert on our current understanding of atherosclerosis.

## Introduction

The classical concept of a “vulnerable plaque” originates from the 1980s and depicts plaque rupture in patients who died from coronary syndromes as the major pathological cause of acute myocardial infarction (1). The concept describes a lipid-rich, atheromatous plaque with a thin fibrous cap and local infiltration of inflammatory cells that cause proteolytic activity and degradation of the stabilizing extracellular matrix (ECM). Genome-wide association studies (GWAS) and lesion-based transcriptomic studies point to a diverse landscape of mechanisms leading to atherosclerotic lesion initiation and progression. Vascular obstructive lesions are characterized by significant variability in pathology-based characteristics. Thrombotic occlusions may arise from traditionally described ruptured vulnerable plaques, but also unruptured eroded plaques and microcalcifications can be complicated by destabilisation (2).

**Figure 1:**
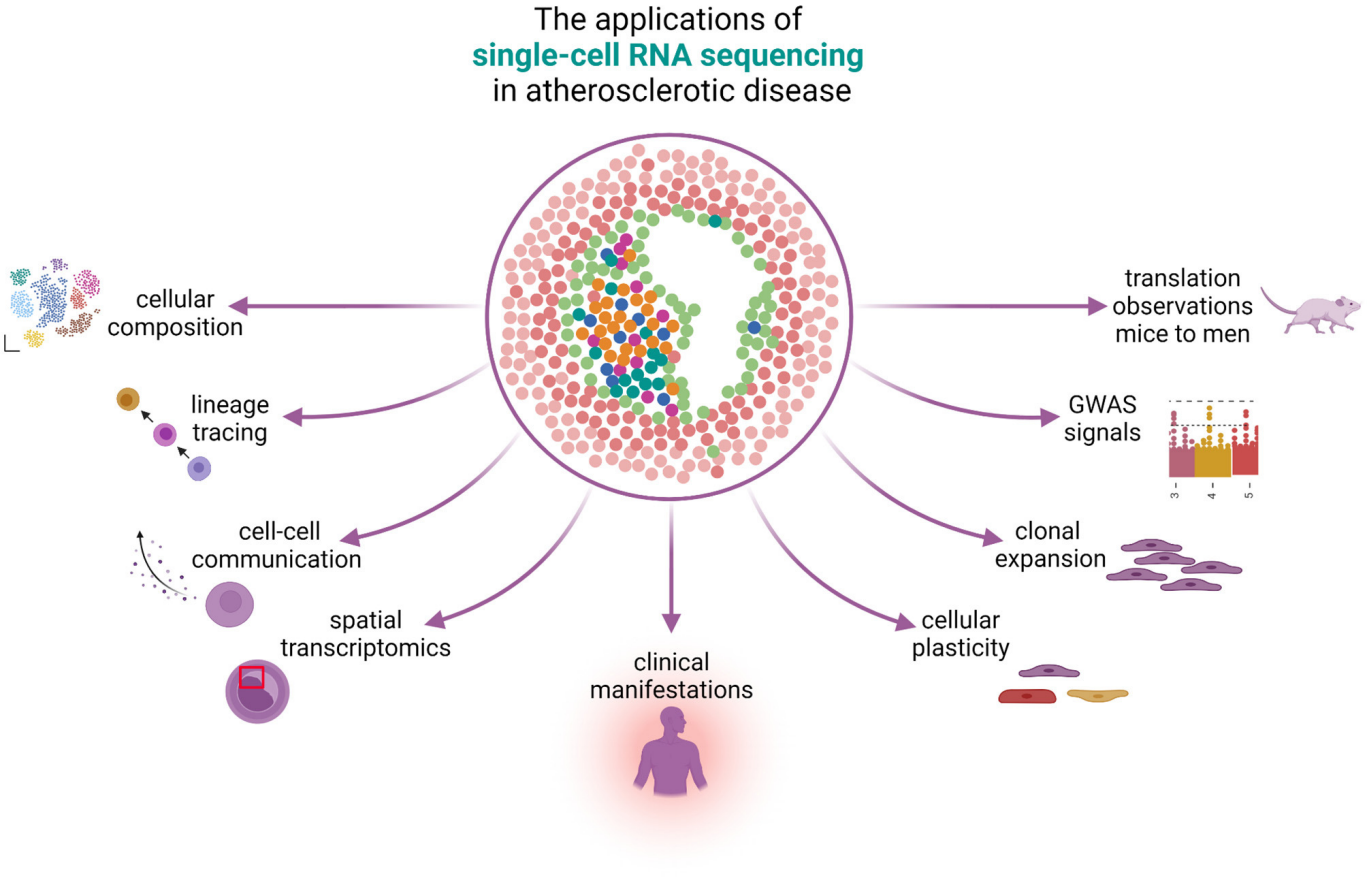
The applications of single-cell RNA sequencing in atherosclerotic disease. scRNA-seq can be used to study many different aspects of atherosclerotic disease. The knowledge on cellular composition can give insights on lineage tracing and can be used to study cellular plasticity, clonal expansion, and reveal cell-gene pairs from GWAS signals. Spatial transcriptomics reveals the physical locations of different cell populations in the plaque and can additionally be used to study cell-cell communication. scRNA-seq can reveal differences in cells under the influence of different clinical presentations and bridge the gap between mice and men.

Bulk micro-array and sequencing of atherosclerotic plaques obtained from human patients (3–6) or animals (7) highlight networks and pathways that could accelerate lesion progression. The heterogeneous nature of the tissue complicates the interpretation and deconvolution of the transcriptomic signal. Therefore, it is difficult to pinpoint which cells are responsible for this signal, and the effect of rare but crucial cells is lost in the noise. Fine-tuning the representation of the cellular composition and concomitant gene expression profiles is facilitated by single-cell RNA sequencing (scRNA-seq). The research field is experiencing a rapid increase in scRNA-seq efforts in the domain of atherosclerosis (7–15). Numerous scRNA-seq datasets are already available for the broad scientific community for reanalysis or *via* online tools like *PlaqView* (16). For both mice and humans, the present cell (sub-)populations have been meticulously described. These studies of atherosclerotic tissue may hold promise in the translation of mouse-to-human findings and provide clues to phenotypic changes in human cell populations that have so far only been described in animal models and cell cultures. In this review, we provide an overview of the findings made with scRNA-seq in atherosclerotic tissue. In addition, we discuss the possible answers that scRNA-seq will potentially provide to long-standing questions that have remained unanswered to date (Figure 1).

### Going Solo

Exploring the cellular diversity of plaques is hindered by tissue heterogeneity. The broad spectrum of cells present in plaque tissue has been unraveled *via* histology, highlighting the classical image of the atherosclerotic plaque with the thin cap comprised of smooth muscle cells (SMCs) and infiltrating immune cells (1). Progress in various –omics techniques has elucidated protein content (17, 18) and transcriptomic content of plaque cells *via* DNA microarrays (3, 4, 6) and bulk RNA-seq (5, 19), providing more insight into the various processes. However, whole tissue (bulk) transcriptomics provides the sum of the transcriptome for all the cells in the tissue and provides no direct information about cell composition. Consequently, the most prevalent cell types dominate the outcomes and subsequent interpretations of bulk transcriptomics efforts, hiding the biology, and heterogeneity of individual cells in their numbers. Each cell type has its unique contribution to plaque manifestation, which is affected by both intrinsic and environmental factors -such as risk factors and medication. Therefore, it is not surprising that the current trend is leaning toward methods that allow for more optimal resolution at the cellular level, evident from the rise in papers using single-cell methods. Flow cytometry and mass cytometry can provide single-cell resolution. However, they are biased as they are dependent on prior knowledge about the phenotype and markers of the cells present in the sample. Predetermined antibodies have to be added to the cell suspension, which can subsequently be analyzed. These methods require in-depth knowledge of the available cell types and sparsely allow for discovering unexpected new or rare cell populations.

In contrast to flow cytometry, scRNA-seq does not require any prior knowledge of cell composition. Rapid developments in the–omics field have benefitted the progression from bulk RNA-seq to single-cell (sc)RNA-seq. The first scRNA-seq effort in 2009 (20) has opened the gates to a rapid increase in applying this technique to various fields, with no exception to atherosclerotic research (8–11, 21, 22). The main advantages are that it can be applied unbiasedly, highlighting new and known cell populations from heterogenic tissue (23). At the same time, they simultaneously uncover the (often subtle) transcriptomic differences created by their unique environment. The rising popularity of scRNA-seq has resulted in efforts to describe the transcriptome of heterogenic tissues and drove the development of bioinformatics tools to analyse the data that follows—ranging from tools to pre-process, analyse and visualize data (24–26), to in-depth specialized follow-up experiments such as identifying rare cell types (27), cell trajectory analysis [evaluated by Saelens et al. (28)], RNA velocity (29) and cell-cell communication [reviewed by Armingol et al. (30)], amongst others. In the following text, we will focus on the discoveries made through scRNA-seq in atherosclerotic research and their implications for plaque characterization. We will discuss the current knowledge on cellular composition of lesions, lineage tracing experiments in mice, cell-cell communication, the importance of sex-stratified research, and examine the technical considerations of scRNA-seq research. For the future perspectives we examine spatial scRNA-seq, the impact of scRNA-seq on pathological specification of atherosclerosis, cell plasticity, clonal expansion, integration of genetic and transcriptomic research, and finally the translation from mice to men.

## Current Knowledge About Cellular Composition: Insights From scRNA-seq

### Cellular Composition in Human and Mouse Plaques

The most significant impact that scRNA-seq has on our understanding of the atherosclerotic plaque is the meticulous dissection of cell populations that are present (8–14)—including multiple types of SMCs, endothelial cells (ECs), and immune cell sub-sets. The cell composition and cell state affect the plaque stability and may lead to different clinical manifestations. The bulk RNA-seq analysis of 654 human lesions uncovered five main plaque types that correlate with symptoms at admission (19). Deconvolution of this bulk data revealed that these plaque types have different underlying cell compositions. Indicating that underlying clinical symptoms can potentially be detected in individual cell populations using scRNA-seq. Here we will briefly discuss findings on the major cell populations of lesions reported in mice and humans. More comprehensive descriptions of the cellular landscape of atherosclerotic lesions are discussed elsewhere (31–33).

The vascular SMCs are located in the vessel wall. They provide structural support and regulate blood flow and blood pressure. Although vascular SMCs are omnipresent in the vasculature, in atherosclerosis, they exhibit distinct phenotypes. In human plaques, we (10) found *two* major subclasses of SMCs, one with contractile and the other showcasing synthetic characteristics. The synthetic phenotype showed low expression of typical SMC markers and upregulation of ECM genes, suggesting that these cells are cap-derived. In a recent review, authors challenge the black and white division between contractile and synthetic SMCs in the vasculature (34). Pan et al. (11) report SMCs (corresponding to a contractile phenotype), fibrochondrocytes, and an intermediate cell population named the intermediate cell state. They also report the presence of fibroblasts in carotid arteries. In aortic arteries and mice, Wirka et al. (8) report fibroblasts and phenotypically modulated SMCs. The value of scRNA-seq in unraveling the fate and origin of transdifferentiating SMCs is underlined by the association of *TCF21* expression with the phenotypic switch of SMC into fibrocytes. With the subsequent strengthening of the fibrous cap and stabilization of the lesion (8).

A thin layer of ECs coats the inner layer of the artery, forming a barrier between the lumen and the artery. Endothelial dysfunction aggravates vascular diseases such as atherosclerosis (35). The role of these cells in disease progression ranges from athero-protective to inflammatory. Our group (10) and others (11) report two main distinct EC populations. Hidden within are also cells undergoing active angiogenesis or those that display the signs of epithelial to mesenchymal transition (10).

The infiltration of inflammatory cells into the intima is a hallmark of disease progression. Macrophages (Mϕs) can be observed during all phases of atherosclerotic lesion formation. Monocytes are rapidly recruited into the lesion and can differentiate into Mϕs or dendritic cells. In a recent meta- analysis (31), authors discriminate between five sub-populations of Mϕs in murine atherosclerosis: resident-like macrophages, foamy Trem2 macrophages, inflammatory macrophages, IFNIC macrophages, and cavity macrophages. Of note, not all Mϕs found in lesions are of monocyte origin (36, 37). In human lesions, Fernandez et al. (9) reported classically activated M1 macrophages. Further transcriptional analysis identified four different Mϕ populations with distinct functions. They reported activated Mϕs, and a population expressing inflammatory genes, and an MMP inhibitor, which could impair ECM degradation and subsequently a more stable plaque. The third population was pro-inflammatory, and the final population showed the transcriptional signature of foam cells with anti-inflammatory signaling. We (10), found three Mϕ populations. Two of which were pro-inflammatory and expressed *IL-1*β or *TNF*. The third population was foam cell-like and exhibited a fibrosis promoting phenotype. This population also showed alpha-actin expression, which potentially indicates a shift to SMC or from SMC to Mϕs. Cochain et al. (13) reported three Mϕ populations in mice. The first population represented a resident-like Mϕ, which is also present in healthy aortic tissue. The other two populations were atherosclerosis specific. The presence of these Mϕ populations was confirmed in human tissue (10).

T cells exhibit significant heterogeneity within the plaque. Fernandez et al. (9) reported that T cells in the plaque demonstrate cytotoxicity, activation, and exhaustion, whereas T cells in the blood circulation show cytokine inhibition, active RNA synthesis, and metabolic reprogramming. They also reported that T cells from symptomatic patients differ from symptomatic patients. However, the number of patients used is small (*n* = 3). Similarly, we (10) found a diverse landscape of CD4^+^ and CD8^+^ T cells populations, the main difference being their activation state. The subclass phenotypes varied from cytotoxic to more quiescent. scRNA-seq of plaques has established the existence of unforeseen T cell clusters with mixed Th1 and Treg cell transcriptional programs such as ApoB reactive T cells (38). In mice, single-cell RNA-sequencing of plaque immune cells revealed differential expression patterns of Tregs in progressing vs. regressing plaques (39).

The spectrum of cell populations uncovered with scRNA-seq is complex and, without a doubt, still incomplete. The community will significantly benefit from a unified nomenclature of cell populations since transcriptome-based names of cell populations vary across multiple studies (40). The aggregation of this knowledge would simplify further research into understanding the molecular mechanisms in atherosclerosis.

### Lineage Tracing

The cells in human atherosclerotic plaques have long been considered fully differentiated and non-plastic. However, *in vitro* and mice experiments have revealed the potential of vascular cell types to undergo phenotypic switching (a process also called dedifferentiation or phenotypic modulation) (36, 41, 42) and revealed the enormous differentiation potential of vascular cells. Endothelial cells can become mesenchymal cells by a process called EndoMT, and SMCs can undergo phenotypic modulation and differentiate into Mϕ-like, fibroblast-like, osteoblast-like, or chondrocyte-like cells. EndoMT can occur under the influence of disturbed blood flow (43, 44) and strongly affect the number of SMCs that stabilize a fibrous cap (45). However, the role and existence of EndoMT in disease is not without controversy (46). When the mechanisms are clear, the current definition of an atherosclerotic plaque eventually can be fine-tuned or altered. Determining the transcriptomic landscape of single cells in human atherosclerotic plaques with scRNA-seq may identify cells in a transitional state, and the trajectory and fate of phenotypic cell switching can be depicted.

**Figure 2:**
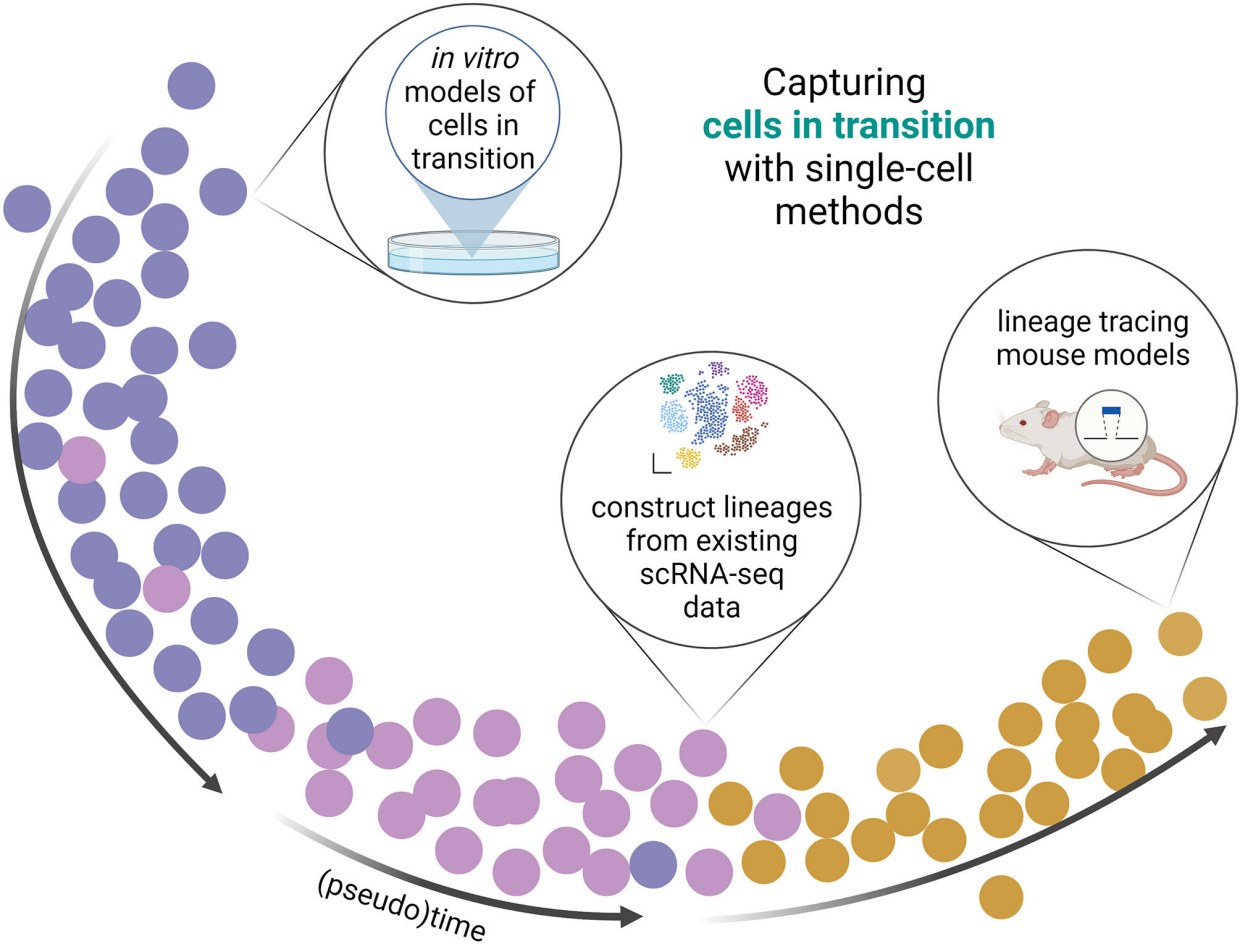
Capturing cells in transition with single-cell methods. Single-cell methods fascilitate the detection of cells in transition. scRNA and/or scATAC facilitate (re)constructing or confirmation of lineages of cells transitioning between phenotypes or cell states. With bioinformatical tools, data from *in vitro, in silico*, and *in vivo* experiments can be used to place cells on an artificial timeline (pseudotime) from which the cell transitions can be studied.

One of the particular advantages of using mouse model systems is the ability to trace cells over time, providing a unique viewpoint on atherosclerotic lesion progression (47, 48). This is not directly possible in humans but can be inferred using the appropriate methods. For example, previous attempts at human lineage tracing involved the tracing of histone modifications on histological slides (49). Human atherosclerotic research is hampered by a cross-sectional design, but the (temporary) preservation of transcriptomic features may point to cells in transition. In addition, bioinformatics tools such as the inference of cell trajectories, also known as “pseudotime” may facilitate the capturing of cells in transition. Pseudotime is a computational technique used to determine the pattern of a dynamic process experienced by cells and then arrange the cells based on their progression through the process. Differences in gene expression between cells result from dynamic processes such as simple cell cycle progression or cell differentiation. In pseudotime such differences are depicted by placing cells along a continuous path that represents the evolution of the process rather than dividing cells into discrete clusters. To find evidence of EndoMT or phenotypic switching in heterogeneous tissue is not unambiguous, but knowledge from lineage tracing experiments in mice can be used to impute this (Figure 2). The assumption then is, that these processes are continuously active and cells from transient stages are always present. However, mice lesions are harvested in an earlier stage of atherosclerosis, whilst human plaques have progressed much further and are end-stage, often symptomatic, lesions. Naturally occurring markers fluctuate during cell transitions and cell fates are harder to trace without a reporter gene. For this reason, it is possible that the intermediate stages are not captured in either case and it is difficult to determine if they were present at all. Lineage tracing is a powerful tool to investigate putative cell transitions in time. A common practice is to label individual cells at an early or induced time point to trace the lineage of labeled daughter cells at a later time point. Lineage tracing is particularly insightful when the process at hand is less unambiguous to impute, such as phenotypic switching. The limitations of this analysis are reviewed elsewhere (50).

The fate of bone marrow derived cells can be followed in mice *via* bone marrow transplant. This method is often combined with lineage tracing techniques where the labeled bone marrow is grafted into wild-type or sex-mismatched mice. These studies show that bone marrow derived cells mainly give rise to inflammatory cells in the plaques and do not transdifferentiate into SMCs or ECs (51). However, supportive scRNA-seq evidence is lacking. A lineage tracing study following the fate of myeloid cells revealed that specialized aortic intima resident macrophages reside in the intima of the aortic arch (52). These resident macrophages give rise to early foam cells of the lesion but were not sustained during atherosclerotic progression. FoxP3 lineage tracing mice experiments showed that Treg cells switch to pro-atherogenic T follicular helper cells during atherosclerosis (53, 54). In order to determine which cells in plaque have SMC origin and how these cells contribute to disease, Shankman et al. (47) used an SMC *YFP*^+/+^*Apoe*^−/−^ mouse model and labeled medial SMCs with YFP labels after tamoxifen injection. This revealed that 36% of *LGALS3*^+^ cells were of SMC origin and rather than of myeloid origin. Furthermore, they state that around 80% of cells with SMC origin are phenotypically modulated. A study from the same group with SMC^−*DualLineage*^ tracing mouse models showed that the percentage of *LGALS3*^+^ cells was lower at around 25% (45). scRNA-seq data from these mouse models made it possible to compare cells across species. This revealed that all cell populations observed in the mice could be identified in human data, indicating that the transcriptional signatures can be used to identify cells that cannot be lineage traced in humans. The tamoxifen-induced *ROSA26*^*ZsGreen*1/+^; *Myh11-CreER*^*T*2^ mouse model of Pan et al. (11) revealed that SMCs in the lesion give rise to multiple cell-populations or cell states, including fibrochondrocytes and Mϕ-like cells. In a recent meta-analysis of scRNA-seq and scATAC-seq lineage tracing experiments, Conklin et al. (40) conclude that lipid loading of SMCs *in vivo* does not correlate to the changes observed *in vitro*. Highlighting the added value of lineage-tracing experiments compared to *in vitro*.

### Cell-Cell Communication

Cell-cell communication (CCC) and cell-cell interactions are interactions between cells regulated by biochemical signaling. Molecules excreted by cells can be used to establish CCC or are part of the structural integrity of tissue, such as the extracellular matrix. scRNA-seq offers the opportunity to study the communicative links between cells in the lesions. Although a proxy for actual protein-protein interactions, the detailed RNA profile of cells can be leveraged to infer CCC from a transcriptomic viewpoint. The bioinformatical tools to impute CCC from this data have been rapidly maturing [reviewed by Almet et al. (55)], varying in their sensitivity and approach.

Previously, we examined the potential receptor-ligand interactions in carotid lesions. Overall, the majority of the interactions were between myeloid cells, SMCs, and ECs (10). The low number of potential interactions between immune cells by the lack of T Cell receptor-related genes can be possibly accounted to their absence in the receptor-ligand interaction database. The occurrence of false-negative or false-positive communication is not just a result of incomplete databases. For a large part, cells can communicate over a limited spatial distance, limiting the number of interactions that can physically take place within the plaque. Interactions by secreted molecules such as cytokines can, indeed, facilitate communications over larger distances. However, interactions where both ligand and receptor are present on the surface of the cells, such as the interaction between CD36 and THBS1, require physical proximity. This spatial information is lost with regular scRNA-seq. A novel sequencing technique makes use of doublets occurring in scRNA-seq data leveraging the idea that cells that are stuck together are likely to be physically interacting. By deconvoluting the single counterpart of doublet cells, the relationship between the physically interacting cells can be studied (56).

### Single-Cell Sequencing and Understanding of Sex Differences in Atherosclerotic Disease

Next to the well-known risk factors for atherosclerotic disease [e.g., diabetes, smoking, hypertension, hypercholesterolemia, ethnicity (57) etc.] (58) also sex (59, 60) strongly associates with different clinical presentations and underlying atherosclerotic pathology. Understanding the underlying molecular and cellular mechanisms remains a significant challenge.

In murine *BXH ApoE*^−/−^ model, the lesion size was increased in females (61). In humans, the sex-stratified gene regulatory networks derived from bulk RNA-sequencing data from atherosclerotic aortic root tissue showed large sex specific differences (62). The genes involved in female-specific networks have higher expression in SMCs, ECs, and Mϕs and are involved in epithelial to mesenchymal transition, KRAS signaling, and estrogen response, respectively. The next step will be to assess the cell types that are responsible for this transcriptomic diversity between sexes. This highlights the importance of sex stratification in studying risk factors.

Sex-specific atherosclerotic murine (12–14) or multiple samples of both sexes human scRNA-seq data (10) is available. However, for these analyses, a sufficient number of samples is required to reach statistical power and based on lessons from histopathological biobank studies, hundreds of individual samples might be required. Intuitively, this explains the limited amount of studies addressing this topic. It is not unlikely that the scRNA-seq field will face a *déjà vu* when researchers start merging data sets, just like the ongoing GWAS efforts to reach sufficient power for stratified analyses. If sample numbers increase, there are no doubt that the exploration of cell-based gene transcriptomes in humans may resolve many unanswered questions regarding the underlying mechanisms that explain the complex diversity and the mechanisms of how risk factors like sex modify the disease progression.

### scRNA-seq Observations Associate With Hemodynamics

Blood flow is of major influence on the formation of atherosclerosis. Lesions develop primarily in vessel regions where blood flow is disturbed by branching or curvature of the artery. Disturbed flow affects the behavior of arterial cells, but how it affects their respective transcriptome was poorly investigated. In a study on flow effect, Andueza et al. (43) performed scRNA-seq and the single-cell assay for transposase accessible chromatin sequencing (scATAC-seq) in a partial carotid ligation mouse model. Trajectory analysis showed that ECs are capable of dramatic phenotypical changes. The already heterogeneous EC population was found to be transitioning to a mesenchymal, hematopoietic stem cell, endothelial stem/progenitor cell, and unexpected immune cell-like phenotypes (43).

Similarly, Li et al. (44) performed scRNA-seq on mice lesions after partial carotid ligation. Compared to the laminar flow condition, they found cell populations specific for both models. A disturbed flow EC population was found to be more enriched in processes such as epithelial to mesenchymal transition and TGF-β signaling. Disturbed flow-specific SMC and Mϕs were also reported (44). Current studies rarely take into account the influence of flow on the outcome of scRNA-seq experiments. However, with the notion that most studied plaque locations are subjected to disturbed flow (carotid artery and aortic arch), it is reasonable to assume that the disturbed flow could influence expression patterns of EC populations found in these lesions. In the future, spatial transcriptomics can help to resolve these questions.

### Technical Considerations and Limitations

#### Source of Lesions

Atherosclerotic plaques develop in coronary, carotid, cerebral, aortic, iliac, and femoral arteries. The most commonly studied human plaques are located in the coronary and carotid arteries (32, 63). When it comes to defining the plaque on a cellular level, location matters (64). Although the arterial systems share characteristics in plaque morphology, there is a difference is the underlying substrate of the ischemic event. In coronary arteries, a local thrombosis caused by either plaque rupture or erosion leads to ischemia of downstream tissue. In the cerebral circulation, an embolization may take place from a plaque upstream of the cerebrovascular occlusion. In the carotid artery, intraplaque bleedings are observed more frequently, and in a significant number of cases, a fibrous lesion underlies the thrombotic event (65, 66). Human coronary plaque samples availability relies on the use of donor's hearts from recipients during transplantation. On the other hand, carotid plaques are dissected from living patients that can still be asymptomatic at the time of surgery. In murine research, aortic tissue is the most prevalent source (32, 63). Although these differences make comparing the coronary and carotid plaque and interspecies comparisons difficult (12), scRNA-seq can potentially unravel differential processes underlying a clinically relevant event. The common hurdle is tissue preservation prior to sequencing. In order to get the full potential from the lesion, sample handling has to be quick to keep it as fresh as possible, in contrast to histological samples where there is less time constraint for processing.

During carotid endarterectomy, the intima with the plaque is removed from the vessel while the tunica media stays majorly intact. Plaques derived from the aorta are likely clippings of diseased samples taken after heart transplants. This is ultimately reflected in the scRNA-seq data, where aortic plaques often show more significant SMCs numbers and phenotypic variety (8). Cochain et al. (13) observed that macrophages are the most dominant immune cell type in atherosclerotic plaques by scRNA-seq. But researchers using CD45^+^ positive selection with FACS will not report CD45^−^ populations such as SMCs (9). Different digestion protocols can heavily skew cell selection and survival before sequencing, even if there is no prior selection. This can dramatically affect cells with poor survival rates *ex-vivo*, such as foam cells and neutrophils. This way, cell populations can become over and under-represented in scRNA-seq (40, 67). Therefore, caution is needed when interpreting results, and reports of cell ratios should always be carefully considered. Additionally, because of the need for digestion of solid tissues prior to scRNA-seq, the spatial location of cells is lost during the process.

#### Technical Limitations

A major limitation of scRNA-seq is the biases in transcript coverage and low capture efficiency, which additionally vary between protocols (33, 68, 69). Incomplete reverse transcription during second-strand synthesis and amplification of the samples can lead to fewer transcripts with the complete 5′ and/or 3′ ends. Gene length influences efficacy, and shorter genes generally have lower counts in single-cell sequencing compared to bulk RNA sequencing (70). As a consequence, genes with a low expression may not be detected due to dropouts and potentially create false negatives. A related obstacle is the use of poly(T) primer, resulting in only RNAs with a poly-A-tail being sequenced (68). Sequencing non-polyadenylated RNAs would be beneficial since these RNAs often serve regulatory functions.

The cost of scRNA-seq is another limiting factor in comparison to traditional bulk sequencing. Even though since 2017, the costs of single-cell sequencing have dropped significantly, single-cell sequencing is still 10 to 200 times more expensive per sample than bulk sequencing (52). Details on different methodologies are reviewed elsewhere (52).

A natural consequence of the growing popularity of scRNA-seq is the increasing need for specialized experimentalists, developers, and bioinformaticians as scRNA-seq gains popularity. However, finding the right tools to implement scRNA-seq into our research is not without caveats for experienced researchers. For example, the scRNA-tools database tracks 1,124 specialized tools for scRNA-seq analysis across 30 categories at the time of writing (71).

## Future Perspectives

### Spatial Location

One of the current limitations of single-cell transcriptomics is that spatial information of cells is lost, but therein also lies an opportunity for the future. Not only can spatial context provide insight into the mechanisms of atherosclerotic disease, but it also greatly influences our interpretation of cell-cell communication. Multiple ways can be used to visualize the spatial gene expression in plaque tissue. Spatial barcoding, *in situ* hybridization, and *in situ* sequencing can all visualize spatial gene expression. However, none of these techniques can cover the full spectrum of the transcriptome like RNA-seq can provide [reviewed by Longo et al. (72)]. Laser-capture microdissection coupled with RNA-seq and downstream analysis can give a detailed view of specific regions of interest like plaque cap or pinpoint to cells with specific phenotypes, under the condition that these cells can be located. This method has been applied successfully in the past on mouse tissue (73). However, the technical challenges to keep plaque tissue in a condition where it is possible to preserve, locate, isolate, and sequence cells of interest are limiting. The growing interest in these techniques is already reflected in implementing these techniques in the major sequencing platforms (74) and data analysis tools (71, 75). However, the current state of spatial technology sequencing does not fully allow for sequencing at individual cell resolution. Future advancements in spatial sequencing can possibly make it a powerful tool to shine new light on spatio-temporal gene expression in atherosclerotic disease.

### Underlying Mechanisms Explaining the Diversity in Pathological Substrates of Clinically Relevant Plaques

One of the most prominent dogmas in atherosclerotic research is the concept of plaque rupture vs. plaque erosion. A concept well-described through histology and clinical presentation. The atherosclerotic plaque may rupture, which leads to red thrombosis due to blood coagulation components getting access to the core of the plaque. If the thrombus arises in coronary arteries or embolises the cerebral arteries, it results in a myocardial infarction or stroke. Plaques that rupture often have a large lipid core with a thin and weak fibrous cap and are called vulnerable plaques or thin-capped fibroatheromas (TCFAs). Besides plaque rupture, plaque erosion may arise (76, 77). These plaques are rich in ECM, have a thick cap and have little lipid deposits with superimposed (white) thrombi. The characteristics described in the TCFA model serve to date as the surrogate endpoints for a vulnerable plaque in animal models. Among other processes, this has led to valuable insights into the role of LDL oxidation, plaque hemorrhage, accumulation, SMC migration, proliferation and apoptosis, senescence, and the role of the variation of local inflammatory cells in the diseased vessel wall. The mechanisms leading to plaque erosion are less well-understood and are one of the major upcoming challenges of the vascular biology community. Animals models hardly develop a thrombotic response, and the lesion underlying the thrombus is thus far categorized as “stable.” For both processes, the mechanisms on a single cell level have not been revealed. The variety of sub-populations identified could be partly explained by these phenomena. Future research may reveal what cells contribute to a pro-thrombotic micro-environment and provide clues for underlying mechanisms.

### Cell Plasticity

In lesions, cells can switch to a new phenotypical identity. These switches include EndoMT (78) and transition from SMC to a Mϕ-like phenotype or foam cells (36). scRNA-seq allows studying cellular plasticity in atherosclerosis in more detail and higher resolution (79). Tools such as the earlier-mentioned pseudotime can aid in studying these processes in mouse model systems and humans.

Dobnikar et al. (80) looked into the SMCs in healthy vessels and atherosclerotic plaque tissue from mice. A remarkable finding was a distinct SMC cluster that expressed *Sca1*—implying that these cells are transitioning from SMC to a Mϕ-like cell. Indications for the transitional capacity of vascular cells was also demonstrated in mice (45). Three SMC clusters expressed both endothelial cell and SMC marker genes, which suggests a transition between phenotypes. In addition, we found that a small subset of human SMCs was *KLF4*^+^ (10), which points to cells that differentiate into a cell with a more synthetic or Mϕ-like phenotype. The value of scRNA-seq to unravel the fate of and origin of transdifferentiating SMC was underlined by a study by Wirka et al. (8), who found that *TCF21* expression was associated with the phenotypic switch of SMC into fibrocytes, with the subsequent strengthening of the fibrous cap and stabilization of the lesion (8). Another mouse study detected an unexpected cluster of proliferating monocytes with a stem cell-like signature, suggesting that monocytes may persist in a proliferating self-renewal state in inflamed tissue and that not all monocytes differentiate immediately into Mϕs after entering the vascular tissue (15). A recent meta-analysis (40) addressed the SMC heterogeneity between four different murine scRNA-seq datasets. Researchers conclude that SMC are capable of sourcing multiple different trajectories within lesions, with many of these transitioning through a de-differentiated SMC state defined by Pan et al. (11) as stem-cell, endothelial cell, monocyte (SEM) cells. The field would benefit from a coalescent review of plastic cells found in lesions to understand their source and impact on plaque development.

### Attack of the Clones

Clonal expansion theory is derived from cancer research. It is a process where one parent cell multiplies as a consequence of acquired mutations. The notion of clonality in atherosclerosis is not a novel theory (81), but there are doubts on the true monoclonality and its consequence on atherosclerotic cells. The clonal environment is likely already present around the plaque, which contributes to the expansion of the lesion (82). Mice with a *Jak*2^VF^ mutation showed increased inflammatory and proliferative myeloid populations in their lesions, as confirmed with scRNA-seq. In addition, these plaques show more pronounced necrotic cores (83). It is unclear whether these plaques exhibit clonal regions. Clonal expansion is mainly described for leukocytes, but in plaque, the concept applies similarly to SMCs. Mice fed with a high-fat diet showed an increase of dedifferentiated SMCs expressing the stem cell marker *Sca1*, forming hubs of clonally expanded cells that tended to form around the necrotic core. Researchers also report that clonally expanding SMCs aggravate atherosclerosis by activating the complement system (84). scRNA-seq confirmed that these cells exhibit a different phenotype from regular SMCs. Other findings in mice describe sheets of clonally expanded SMCs derived from the media rather than patches (85). The origin of these clonal progenitor cells giving rise to these clonal patches is still unclear, and the occurrence of clonal expansion is seemingly random. Whether or not this clonal expansion of SMCs is beneficial for plaque stability is still up for debate.

**Figure 3:**
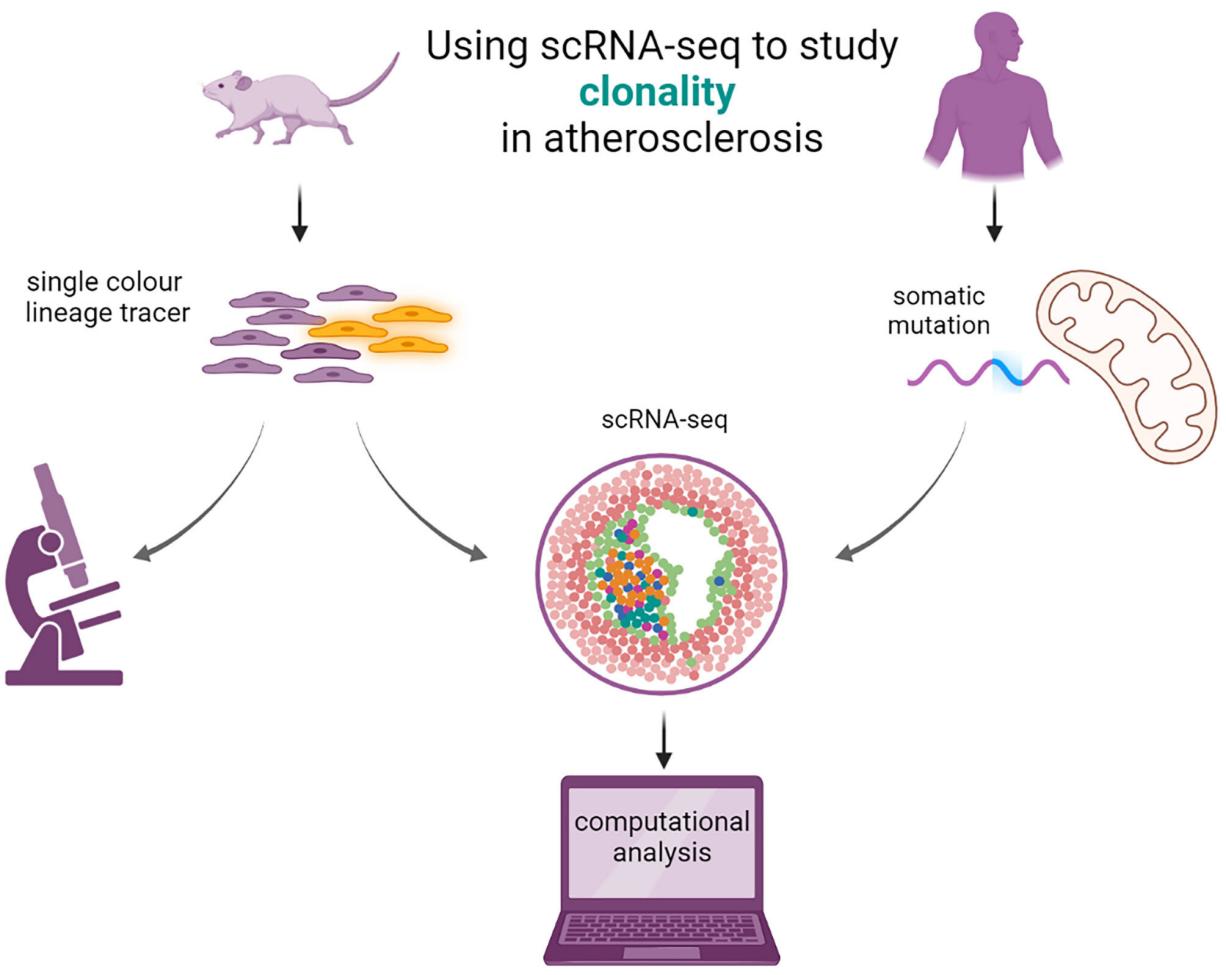
Using scRNA-seq to study clonal cell expansion in atherosclerosis. Single color lineage tracers are added to reporter genes to study the clonality in mice (left). The mice will develop atherosclerosis and patches of clonal cells are visible from microscopy images. scRNA seq can be performed, and computational analysis can elucidate the transcriptomic properties of the cells expressing the lineage tracer. In humans (right), clonality can be imputed from studying the somatic mutations in mitochondria with scRNA- or scATAC-seq from existing data with computational analysis.

The use of lineage tracing techniques has been majorly beneficial for the research on clonality in plaque tissue. A privilege that cannot be extended to human research. The alternatives are tracing naturally occurring markers in know cell differentiation pathways or detecting somatic mutations (Figure 3). A novel approach integrates scRNA-seq and scATAC-seq of mitochondrial RNA for both clonal and lineage tracing (86). This method can be applied to already existing single-cell datasets of lesion tissue and can confirm these long-standing observations of clonal expansion in plaque.

### Integrating scRNA-seq in Post-GWAS Analyses

The reported number of common genetic variants associated with coronary artery disease (87) ischemic stroke (88), and intermediate traits of atherosclerosis, such as carotid intima-media thickness (89), is increasing as the meta-analysis efforts of genome-wide association studies (GWAS) grow more prominent and larger (90). However, the functions of many genes that are considered to be causally related to cardiovascular diseases are unknown for roughly 50% of the loci discovered (90). The expression of these potentially causal genes or other downstream interacting genes can originate from different organs of which the (atherosclerotic) vasculature is one. Much research currently aims to prioritize the “right” genes underlying the GWAS loci through integration with molecular quantitative trait locus (molQTL) analyses in various tissues. In contrast, these efforts prioritize genes but they do not provide information on the cellular context (91). As we (92) and others (21) recently showed, the integration of GWAS summary statistics with plaque-derived scRNA-seq and/or snATAC-seq data aids in the prioritization of genes and cell populations relevant to disease, which in turn can be used as a guideline for *in vitro* and *in vivo* mechanistic research.

This could further be augmented by single-cell molQTL (sc-molQTL) analyses. Indeed, heritable genetic effects that modulate expression at a single-cell resolution may affect specific cellular processes in disease (93, 94). Studies focused on discovering genetic effects on proximal genes, *cis*-acting single-cell expression QTLs (*cis*-sc-eQTLs), across populations will face two challenges. First, most analytical tools are not designed to consider the sparsity (the zero-inflated expression) in single-cell data. New methods are developed, but this field has not matured (95). Second, expression and genetic variation is also population-specific, and many datasets are now mainly derived from European ancestral populations. An inclusive approach to population diversity will provide a fine-grained map of genetic variation affecting cellular processes at a single cell level in disease tissue.

### From Mice to Men

The extrapolation of results from atherosclerotic mouse models to human advanced stages of atherosclerosis is still an ongoing matter of debate. Indeed, the translation from mouse to human regarding the mechanisms of initiation and progression of atherosclerosis has been shown to be complex. Small-scale comparison between specific murine and human cell-subsets have been attempted (8, 10, 11, 52), and to a smaller extent, larger scale comparison efforts have been made (16, 40). However, animals models hardly develop a thrombotic response, and the lesion underlying the thrombus is thus far categorized as “stable.” Nonetheless, the determinants of end-stage atherosclerosis in humans have been used as benchmarks in atherosclerotic mice. However, verification in human plaques was mainly performed by demonstrating gene or protein expression differences between symptomatic and asymptomatic plaques (9). This single gene/protein cross-sectional approach was difficult to translate into a specific role in either plaque stabilization or destabilization. While still observational, scRNA-seq could indicate in which cell a gene is expressed, given the transcriptomic context in which the gene is located. This location is not limited to cell-population-specific expression but extends to their position in networks and pathways. This could provide the opportunity to better translate and verify the value of the mouse models to human plaques. Further comparative research will reveal the extent to which the gene expressions found occurring from a genetically identical mouse strain can be translated to the genetically heterogeneous human population and whether expression patterns are generic or individual-specific. A recent study compared mice and human vascular SMCs and reported that the many similarities favor the continued use of mice models to extrapolate the fate of the human counterpart cells (40). However, in a systematic review, we reported that genes involved in human genetic variants for coronary artery disease, large artery ischemic stroke, or atherosclerotic plaque characteristics seldom show associations in mice (96). The vast body of work describing the cellular composition of mice compared to human findings offers the opportunity to investigate population similarities and could contribute to the translation from murine to human atherosclerosis.

## Concluding Remarks

The value of scRNA-seq in elucidating the mechanisms of arteriosclerosis is beyond doubt. The current literature already indicates a much greater diversity of inflammatory and resident cell types than previously believed. The plasticity of vascular cells has been demonstrated thanks to scRNA-seq, and the first steps to spatial gene expression in plaque are taking place. However, the field would benefit from a consensus in cell-population nomenclature to match findings from different groups and facilitate inter-species comparisons. The rapid development of scRNA-seq will drive down the prices and therefore, more samples can be analyzed, which is currently the biggest limitation. A new limitation will arise, which is inevitably tied to the ever-growing popularity of big data: the expert (man) power to analyse and biologically interpret the data.

## Author Contributions

LS, DT, and GP prepared the original draft. LS drafted the manuscript and designed figures. SL and MM contributed to manuscript extension and revision. All authors contributed to the article and approved the submitted version.

## Funding

This work was supported by grants from: Fondation Leducq (PlaqOmics 18CVD02) to GP. SL was funded through grants from the Netherlands Cardiovascular Research Initiative of the Netherlands Heart Foundation (CVON 2011/B019 and CVON 2017-20: Generating the best evidence-based pharmaceutical targets for atherosclerosis [GENIUS I&II]). We are thankful for the support of the ERA-CVD program druggable-MI-targets (grant number: 01KL1802) and the EU H2020 TO_AITION (grant number: 848146).

## Conflict of Interest

The authors declare that the research was conducted in the absence of any commercial or financial relationships that could be construed as a potential conflict of interest.

## Publisher's Note

All claims expressed in this article are solely those of the authors and do not necessarily represent those of their affiliated organizations, or those of the publisher, the editors and the reviewers. Any product that may be evaluated in this article, or claim that may be made by its manufacturer, is not guaranteed or endorsed by the publisher.
